# Multisynchronization of Chaotic Oscillators via Nonlinear Observer Approach

**DOI:** 10.1155/2014/935163

**Published:** 2014-01-21

**Authors:** Ricardo Aguilar-López, Rafael Martínez-Guerra, Juan L. Mata-Machuca

**Affiliations:** ^1^CINVESTAV-IPN, Avenida Instituto Politécnico Nacional 2508, 07360 San Pedro Zacatenco, DF, Mexico; ^2^Academia de Mecatrónica, UPIITA-IPN, Avenida Instituto Politécnico Nacional 2580, Barrio La Laguna Ticomán, Gustavo A. Madero 07340, México. DF, Mexico

## Abstract

The goal of this work is to synchronize a class of chaotic oscillators in a master-slave scheme, under different initial conditions, considering several slaves systems. The Chen oscillator is employed as a benchmark model and a nonlinear observer is proposed to reach synchronicity between the master and the slaves' oscillators. The proposed observer contains a proportional and integral form of a bounded function of the synchronization error in order to provide asymptotic synchronization with a satisfactory performance. Numerical experiments were carried out to show the operation of the considered methodology.

## 1. Introduction

The synchronization phenomenon of dynamical systems is important in applications, in technology, and has a wealth of science. As it is well known, it is common to associate synchronization phenomenon with periodic signals, in the last years has been realized that chaotic systems can be also synchronized. This has led to several engineering applications; the most important one is related with the information transmission using chaotic systems [1–3].

Clear knowledge of chaos synchronization and dynamical mechanisms behind opens new opportunities both for applications of chaotic signals in engineering and for understanding functionality of electronic circuits, chemical reactions, fluid mechanics, and biological networks, where irregular dynamics occur naturally [4, 5].

Pecora and Carroll [6] introduced the idea of synchronization for possibly chaotic dynamical systems. The synchronization phenomena are concerned with two identical systems, which can be coupled in such a way that the solution of one always converges to the solution of the other, independently of the initial conditions and parameters. However, although one system responds to the other, the reciprocal does not happen and this phenomenon is named master-slave synchronization.

Several applications of master-slave synchronization include the control of chaos and chaotic signal masking, where several methodologies have been considered for synchronization purposes [7–9]. In general, the synchronization of dynamic chaotic oscillator has been realized via two main approaches; the design of feedback controllers to tackle the tracking problem related with the proper synchronization phenomena where oscillators with different order and structure can be synchronized and the design of state observers related with the synchronization of chaotic oscillators with equivalent order and topology. A named dual synchronization of a master oscillator and two slaves systems was carried out for fractional order systems employing a linear-type controller with successes [10].

The main contribution of this paper is to propose a master-slave synchronization scheme, where several slave systems are considered to be synchronized having arbitrary and different initial conditions. In this configuration, the Chen oscillator is considered as the master system and the nonlinear observers are the corresponding slave systems. The idea is that the trajectories of the observers follow the trajectories of the Chen oscillator. The observer structure contains a proportional and integral form of a bounded function of the synchronization error in order to provide asymptotic synchronization with a satisfactory performance.

## 2. The Chen Dynamic Oscillator Model

Chen's dynamical system [4] is described by the following system of differential equations:

(1) $$\begin{array}{c} \Sigma_{m}=\{\begin{array}{c} {\dot{x}}_{1}=\alpha \left(x_{2}-x_{1}\right)\\ {\dot{x}}_{2}=\left(\gamma -\alpha \right)x_{1}-\gamma x_{2}-x_{1}x_{3}\\ {\dot{x}}_{3}=\;x_{1}x_{2}-\beta x_{3}, \end{array} \end{array}$$

where the considered measured signal is assumed as *s* = *x*_2_.

Here, *x*_1_, *x*_2_, and *x*_3_ are the state variables and the parameters *α*, *β*, and *γ* are three positive real constants. This system contains a chaotic attractor when *α* = 35, *β* = 3, and *γ* = 28. The trajectory of the system is specified by (*x*_1_(*t*), *x*_2_(*t*), *x*_3_(*t*)). The singular points (SP) of the system (1) are SP_1_ = (−*ρ*, −*ρ*, 2*γ* + *α*), SP_2_ = (*ρ*, *ρ*, 2*γ* + *α*), and finally SP_3_ = (0,0, 0) where $\rho =\sqrt{\beta \left(2\gamma -\alpha \right)}$.

The divergence of the flow related with the system (1) is as follows:

(2) $$\begin{array}{c} \nabla \cdot ℱ=\frac{\partial f_{1}}{x_{1}}+\frac{\partial f_{2}}{x_{2}}+\frac{\partial f_{3}}{x_{3}}=-\alpha +\gamma -\beta <0 \end{array}$$

when *α* + *β* > *γ*.

Here *ℱ* = (*f*_1_, *f*_2_, *f*_3_) = (*α*(*x*_2_ − *x*_1_), (*γ* − *α*)*x*_1_ − *x*_1_ *x*_3_ + *γx*_2_, *x*_1_ *x*_2_ − *βx*_3_).

Thus, the system (1) is a forced dissipative system similar to a Lorenz system. Thus, the solutions of the system (1) are bounded as *t*⟶*∞*. Chen shows that the system (1) exhibits chaos for specified values of the parameters *α* = 35, *β* = 3, and *γ* = 28.

Let us to consider the system (1) as the master oscillator; in consequence, the slaves oscillators, under the master-slave synchronization scheme, are given by a Chen oscillator model disturbed by external feedback, with the following structure:

(3) $$\begin{array}{c} u=g_{0}\int_{0}^{e}\frac{1}{{z\left(t\right)}^{2}+1}dz+g_{1}e\left(t\right). \end{array}$$

From the above, the corresponding structure of the *i*th slave system is as follows:

(4) $$\begin{array}{c} \Sigma_{si}=\{\begin{array}{c} {\dot{\hat{x}}}_{i1}=\alpha \left({\hat{x}}_{i2}-{\hat{x}}_{i1}\right)\\ \;\;+g_{0}\int_{0}^{e_{i}}\frac{1}{{z\left(t\right)}^{2}+1}dz+g_{1}e_{i}\left(t\right)\\ {\dot{\hat{x}}}_{i2}=\left(\gamma -\alpha \right){\hat{x}}_{i1}-\gamma {\hat{x}}_{i2}-{\hat{x}}_{i1}{\hat{x}}_{i3}\\ \;\;+g_{i0}\int_{0}^{e_{i}}\frac{1}{{z\left(t\right)}^{2}+1}dz+g_{i1}e_{i}\left(t\right)\\ {\dot{\hat{x}}}_{i3}=\;{\hat{x}}_{i1}{\hat{x}}_{i2}-\beta {\hat{x}}_{i3}\\ \;\;+g_{i0}\int_{0}^{e_{i}}\frac{1}{{z\left(t\right)}^{2}+1}dz+g_{i1}e_{i}\left(t\right). \end{array} \end{array}$$

For *i* = 1,2,…, *n*.

## 3. Synchronization Methodology

Now, consider the below general representation of a class of nonlinear system, as the chaotic oscillator:

(5) $$\begin{array}{c} \dot{x}=f\left(x,u\right),\\ s=h\left(x\right)=Cx, \end{array}$$

where *x* ∈ ℝ^*n*^ is the variable states vector, *u*∈ℝ^*m*^ is the control input, *m* ≤ *n*, and *s* ∈ ℝ is the corresponding measured signal; *f*(*x*, *u*) is a differentiable vector function such that *f*: ℝ^*n*^ × ℝ^*m*^⟶ℝ^*n*^ it is Lipschitz continuous (with Lipschitz constant, *ℋ* = sup⁡_*u*∈ℝ^*m*^, *x*∈*Ω*⊂ℝ^*n*^_ | *f*′(*x*, *u*)|≥0, being *Ω* a compact set,) if and only if it has bounded first derivative; one direction follows from the mean value theorem as follows:

(6) $$\begin{array}{c} ||f_{i}\left(x,u\right)-f_{i}\left(\hat{x},u\right)||\leq ℋ\left(x_{i}-{\hat{x}}_{i}\right). \end{array}$$

**Proposition 1**

The dynamic systems (7) act as slave systems for the system (1)

(7) $$\begin{array}{c} {\dot{\hat{x}}}_{i}=f_{i}\left({\hat{x}}_{i},u\right)+g_{i0}\int_{0}^{e_{i}}\frac{1}{{z\left(t\right)}^{2}+1}dz+g_{i1}e_{i}\left(t\right). \end{array}$$

That is to say,

(8) $$\begin{array}{c} \underset{t\to \infty }{\lim}e_{i}\left|\left(t\right)\right|=0, \end{array}$$

with $e_{i}=x_{i}-{\hat{x}}_{i}$, or in another way,

(9) $$\begin{array}{c} \underset{t\to \infty }{\lim}\left|x_{i}-{\hat{x}}_{i}\right|=0, \end{array}$$

where *e*_*i*_ is defined as the *i*th synchronization error.

Note that the nonlinear feedback of the slave systems satisfies the following property:

(10) $$\begin{array}{c} \left|\int_{0}^{e_{i}}\frac{1}{{z\left(t\right)}^{2}+1}dz\right|\leq M<\infty , \end{array}$$

where *M* is a positive constant.

From the above, the dynamic modeling of the *i*th synchronization error is defined employing (1) and (7), as

(11) $$\begin{array}{c} {\dot{e}}_{i}=f_{i}\left(x_{i},u\right)-f_{i}\left({\hat{x}}_{i},u\right)-g_{i0}\int_{0}^{e_{i}}\frac{1}{{z\left(t\right)}^{2}+1}dz+g_{i1}e_{i}\left(t\right). \end{array}$$

### 3.1. Convergence Analysis

Consider the Lyapunov candidate function for the *i*th synchronization error:

(12) $$\begin{array}{c} \ell_{i}=e_{i}^{T}Re_{i}={||e_{i}||}_{R}^{2},\;\;R=R^{T}>0. \end{array}$$

The time derivative along the trajectories of (12) is

(13) ℓi˙=e˙iTRei+eiTRei˙  =(fi(x,u)−fi(x^,u) −gi0∫0ei1z(t)2+1dz+gi1ei(t))TRei     +eiTR(fi(x,u)−fi(x^,u) −gi0∫0ei1z(t)2+1dz+gi1ei(t))=2eiTR(fi(x,u)−fi(x^,u)) −2eiTR(gi0∫0ei1z(t)2+1dz+gi1ei(t)).

(a) The matrix *R* can be expressed as *R* = *ℬℬ* ^*T*^; then
  (14) ||eiTR(fi(x,u)−fi(x^,u))|| =||eiTℬℬT(fi(x,u)−fi(x^,u))||=||ei~Tfi~||,

where ${\tilde{e_{i}}}^{T}={e_{i}}^{T}ℬ$ and $\tilde{f_{i}}={ℬ}^{T}\left(f_{i}\left(x,u\right)-f_{i}(\hat{x},u)\right)$.

Then,

(15) ||e~iT||=(e~iTei~)1/2=(eiTℬℬTei)1/2=(eiTRei)1/2=||ei||R.

As well, it is defined as

(16) $$\begin{array}{c} ||\tilde{f_{i}}||={||f_{i}||}_{R}. \end{array}$$

Hence,

(17) ||eiTR(fi(x,u)−fi(x^,u))|| =||e~iTf~||≤||e~iT||||fi~||=||ei||R||fi||R.

(b) Taking into account (a),
  (18) ||eiTR(gi0∫0ei1z(t)2+1dz+gi1ei(t))|| ≤||gi0∫0ei1z(t)2+1dz+gi1ei(t)||||ei||R.

From (a) and (b) and considering the bounded assumptions given by equations (6) and (10) of the Chen oscillator and the nonlinear feedback, the following is considered:

(19) $$\begin{array}{c} \dot{\ell_{i}}\leq 2\left[{||e_{i}||}_{R}ℋ{||e_{i}||}_{R}-g_{i0}{M||e_{i}||}_{R}-g_{i1}{||e_{i}||}_{R}^{2}\right]. \end{array}$$

Then,

(20) $$\begin{array}{c} \dot{\ell_{i}}\leq 2\left[\left(ℋ-g_{i1}\right){||e_{i}||}_{R}^{2}+g_{i0}M{||e_{i}||}_{R}\right]. \end{array}$$

Backing to the original synchronization error,

(21) $$\begin{array}{c} 2{||e_{i}||}_{R}\frac{d}{dt}{||e_{i}||}_{R}\leq 2\left[\left(ℋ-g_{i1}\right){||e_{i}||}_{R}^{2}+g_{io}M{||e_{i}||}_{R}\right]. \end{array}$$

Simplify

(22) $$\begin{array}{c} \frac{d}{dt}{||e_{i}||}_{R}\leq \left(ℋ-g_{i1}\right){||e_{i}||}_{R}+g_{i0}M. \end{array}$$

Finally solve the above differential inequality

(23) ||ei||R≤||ei||R0exp⁡((ℋ−gi1)t) +gi0Mℋ(1−exp⁡((ℋ−gi1)t)).

Note that for time large enough, the synchronization error lies to a closed ball **B** with radius proportional to *g*_*i*0_ *M*/*ℋ*, which can be diminished by the adequate selection the parameter *g*_*i*0_; then,

(24) $$\begin{array}{c} \underset{t\to \infty }{\lim}{||e_{i}||}_{R}\leq \frac{g_{i0}M}{ℋ}. \end{array}$$

Let us consider the Rayleigh inequality; then,

(25) $$\begin{array}{c} \lambda_{\min}\left(R\right){\underset{t\to \infty }{\lim}||e_{i}||}^{2}\leq \underset{t\to \infty }{\lim}e_{i}^{T}Re_{i}=\underset{t\to \infty }{\lim}{||e_{i}||}_{R}\leq \frac{g_{i0}M}{ℋ}. \end{array}$$

Finally,

(26) $$\begin{array}{c} \underset{t\to \infty }{\lim}||e_{i}||\leq \sqrt{\frac{g_{i0}M}{\lambda_{\min}\left(R\right)\;ℋ\;}}. \end{array}$$

## 4. Numerical Experiments

**Figure 1:**
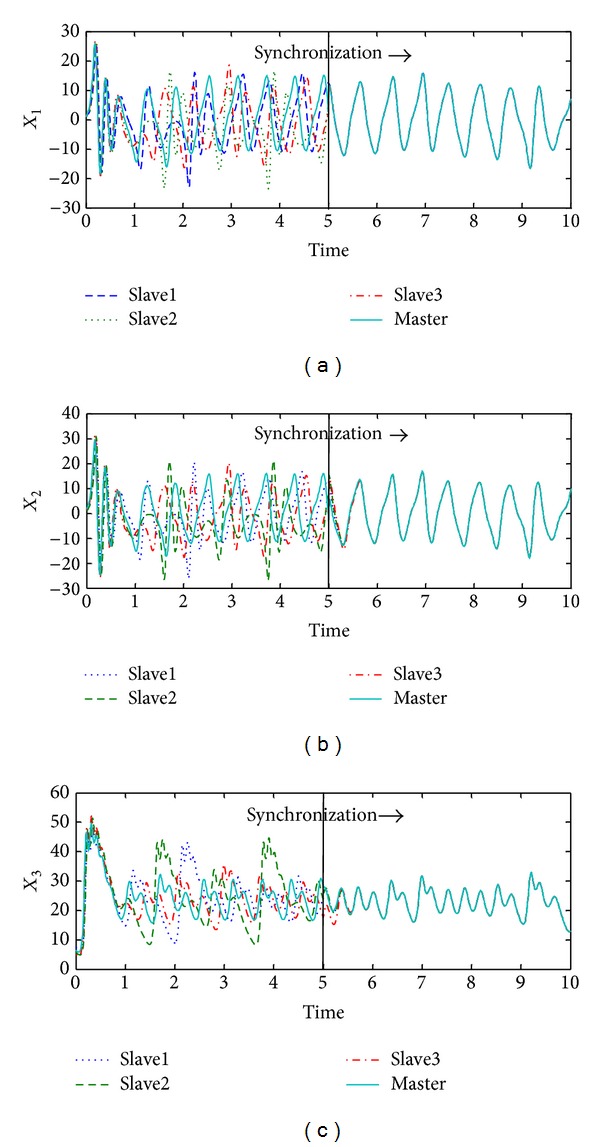
Trajectories of master and slaves systems in synchronization.

**Figure 2:**
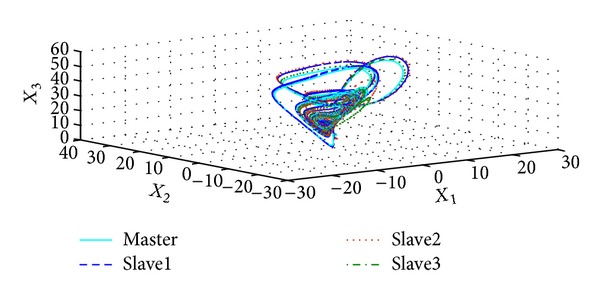
Phase portrait of the master-slaves synchronization.

**Figure 3:**
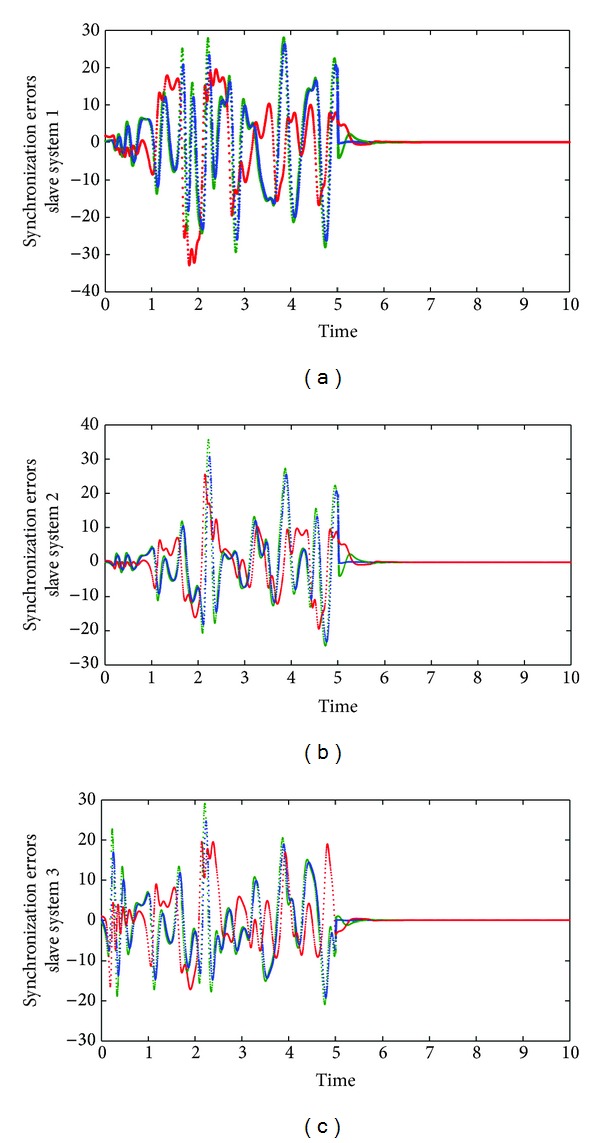
Synchronization errors.

Numerical simulations were done in order to provide the performance of the proposed synchronization methodology; a personal computer (PC) with Intel Core i7 processor and the ode solver from MatLab (ode23s library) were employed. For the master oscillator (1), the following initial condition was considered: *x*(0) = [1.5, 1.25, 7.5] and for the slaves oscillators (7), the corresponding initial conditions were ${\hat{x}}_{i}(0)=[(1.2,1.0,5.8),\;\;(1.8,1.5,5.0)$, (1.25,1.35,7.0)], respectively; the parameters of the master and the slave systems are presented previously in Section 3. The synchronization procedure was turned on at time *t* = 5 s; the vector parameters of the corresponding feedbacks on the slaves oscillators are the same for all considered slaves systems; that is, *g*_*i*0_ = [500, 500, 100] and *g*_*i*1_ = [10,10,2] for all *i* = 1,2,…, *n*, where *n* = 3. Figures 1(a), 1(b), and 1(c) show the variables of the observers (slaves systems), ${\hat{x}}_{i1},\;{\hat{x}}_{i2}$ and ${\hat{x}}_{i3}$, synchronized with the coordinates of the Chen oscillator, *x*_1_,  *x*_2_, and *x*_3_, respectively; it is important to note the fast response of the proposed synchronization methodology which provides short overshoots and settling time in order to reach the coordinates of the master system.  Figure 2 illustrated how the attractors of the master Chen oscillator and their slaves systems are the same, after a time. Finally, Figure 3 gives us the synchronization or estimation errors, which tend to zero for all the considered slaves systems, which is in accordance with the theoretical convergence sketch of proof; this allows concluding the satisfactory performance of the considered methodology.

## 5. Conclusions

In this work, we tackled the master-slave synchronization problem via nonlinear observer design. We considered the Chen oscillator as the master system and the slaves systems were the proposed nonlinear observers, which contains a proportional and integral forms of a bounded function of the synchronization errors to guarantee asymptotic synchronization. A Lyapunov framework is considered to show the stability of the synchronization errors with success. Numerical experiments showed the performance of the proposed methodology.
